# Efficacy of Sacubitril/Valsartan in a Patient With Heart Failure and Impaired Secretion of Atrial Natriuretic Peptide Due to Long-Standing Persistent Atrial Fibrillation

**DOI:** 10.7759/cureus.71844

**Published:** 2024-10-19

**Authors:** Satoshi Kurisu, Hitoshi Fujiwara

**Affiliations:** 1 Department of Cardiology, National Hospital Organization (NHO) Hiroshimanishi Medical Center, Otake, JPN

**Keywords:** echocardiography, electrocardiogram, mitral regurgitation, neprilysin, volume overload

## Abstract

Atrial natriuretic peptide (ANP) is a circulating hormone released from the atria in response to wall stretch and volume overload in the setting of heart failure. When atrial fibrillation (AF) becomes long-standing persistent, ANP secretion in response to volume overload is impaired due to degenerative changes of the atria. Here, we report a case of heart failure with preserved ejection fraction and impaired ANP secretion due to long-standing AF. N-terminal pro-brain natriuretic peptide (NT-proBNP) level was elevated (269 pg/mL), whereas the increase in ANP level was marginal (46.1 pg/mL), suggesting impaired ANP secretion due to long-standing AF. Valsartan (80 mg/day) was replaced with sacubitril/valsartan (100 mg/day) without changing other medications. Administration of sacubitril/valsartan was effective in improving the patient’s symptoms, such as dyspnea and edema, and reducing NT-proBNP level by increasing endogenous ANP level from 46.1 pg/mL to 117 pg/mL in the first four weeks. This case highlights the possibility of impaired ANP secretion in response to volume overload as a predictor of the diuretic effect of sacubitril/valsartan in heart failure. This may lead to individualized treatment for each patient with heart failure based on natriuretic peptide profiles.

## Introduction

Heart failure with preserved ejection fraction (HFpEF) is a multifaceted pathogenesis disease and accounts for nearly half of all heart failure cases [1,2]. Atrial fibrillation (AF) frequently coexists with HFpEF, and the progression from paroxysmal to persistent AF is associated with serious adverse events, including stroke, systemic embolism, and hospitalization for heart failure [3,4]. Atrial natriuretic peptide (ANP) is a circulating hormone released from the atria in response to wall stretch and volume overload in the setting of heart failure. ANP has various effects such as natriuresis, diuresis, vasodilation, and blockade of the renin-angiotensin-aldosterone system, counteracting the volume-overloaded state [5]. When AF becomes long-standing persistent, ANP secretion in response to volume overload is impaired due to degenerative changes of the atria [6-8].

Sacubitril/valsartan, which increases natriuretic peptide levels through the inhibition of neprilysin, has been recently shown to have beneficial effects on heart failure [9,10]. These effects may depend more on ANP than brain natriuretic peptide (BNP) because neprilysin has higher substrate specificity for ANP than BNP [11]. The diuretic effect of sacubitril/valsartan may be promising, especially in patients with heart failure and impaired ANP secretion.

Here, we reported a case of HFpEF and impaired ANP secretion due to long-standing AF. This study aimed to assess the efficacy of sacubitril/valsartan in a patient with heart failure and impaired ANP secretion.

## Case presentation

An 87-year-old woman with persistent AF for more than 15 years, who had been treated with edoxaban (15 mg/day), presented to her primary care clinic with a three-week history of dyspnea and edema in both lower extremities. She had no history of stroke or other embolic events related to AF. She was taking valsartan (80 mg/day), amlodipine (5 mg/day), and hydrochlorothiazide (12.5 mg/day) for hypertension. The patient reported a weight gain of 5 kg and azosemide (60 mg/day) was added. However, the patient’s symptoms such as dyspnea and edema were not fully resolved. She was referred to our hospital for further cardiac evaluation.

On physical examination, her pulse rate was 78 beats/minute, blood pressure was 130/54 mmHg, body weight was 64 kg, and oxygen saturation was 94%. A systolic murmur was detected at the left lower sternal border. Pitting edema was noted in both lower extremities. Laboratory tests revealed anemia with a hemoglobin of 9.0 g/dL and chronic kidney disease with a creatinine level of 1.32 mg/dL (Table 1). Liver and thyroid functions were almost normal. N-terminal pro-BNP (NT-proBNP) (269 pg/mL, reference range: <126 pg/mL) and BNP (43.3 pg/mL, reference range: <18.4pg/mL) levels were elevated, whereas the increase in ANP level was marginal (46.1 pg/mL, reference range: <43 pg/mL), suggesting impaired ANP secretion due to long-standing AF.

**Table 1:** Laboratory findings. At initial presentation, NT-proBNP (269 pg/mL) and BNP (43.3 pg/mL) levels were elevated, whereas an increase in ANP level was marginal (46.1 pg/mL), suggesting impaired ANP secretion due to long-standing AF. NT-proBNP: N-terminal pro-brain natriuretic peptide; BNP: brain natriuretic peptide; ANP: atrial natriuretic peptide; AF: atrial fibrillation

Variable	Initial presentation	26th week	Reference range
White blood cell counts (/μL)	5.5 × 10^3^	6.0 × 10^3^	3.3–8.6 × 10^3^
Red blood cell counts (/μL)	2.87 × 10^6^	3.70 × 10^6^	3.86 - 4.92 × 10^6^
Hemoglobin (g/dL)	9.0	11.7	11.6–14.8
Platelet counts (/μL)	161 × 10^3^	179 × 10^3^	158–348 × 10^3^
Aspartate aminotransferase (U/L)	24	21	13–30
Alanine aminotransferase (U/L)	16	11	7–23
Lactate dehydrogenase (U/L)	230	271	124–222
Creatine kinase (U/L)	209	-	41–153
Total protein (g/dL)	6.1	7.4	6.6–8.1
Albumin (g/dL)	3.3	4.1	4.1–5.1
Blood urea nitrogen (mg/dL)	35.1	26.3	8–20
Creatinine (mg/dL)	1.32	1.33	0.46–0.79
C-reactive protein (mg/dL)	0.49	-	0–0.14
Atrial natriuretic peptide (pg/mL)	46.1	69.8	<43
Brain natriuretic peptide (pg/mL)	43.3	-	<18.4
N-terminal pro-brain natriuretic peptide (pg/mL)	269	176	<126
Thyroid-stimulating hormone (μIU/mL)	4.47	-	0.61–4.23
Free triiodothyronine (pg/mL)	2.57	-	1.68–3.67
Free thyroxine (ng/dL)	1.40	-	0.70–1.48

**Figure 1:**
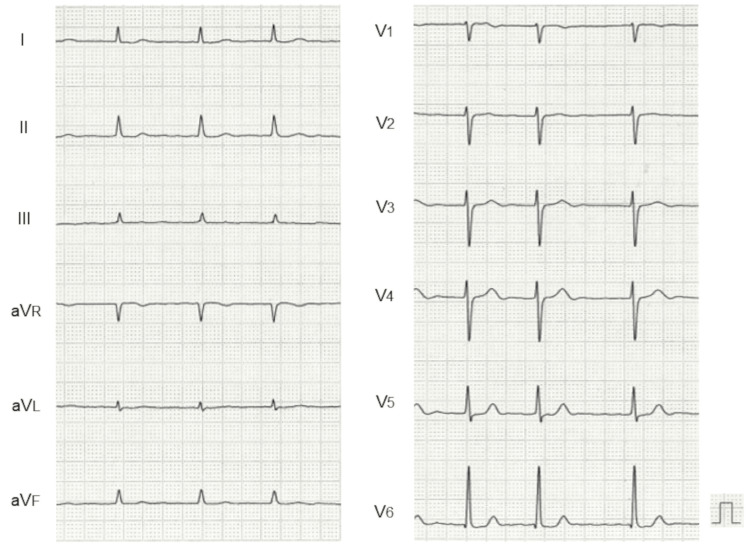
Electrocardiogram. An electrocardiogram revealed AF with fibrillatory wave amplitudes <0.1 mV, the so-called fine AF. AF: atrial fibrillation

An electrocardiogram revealed AF with fibrillatory wave amplitudes <0.1 mV, so-called fine AF (Figure 1).

**Figure 2:**
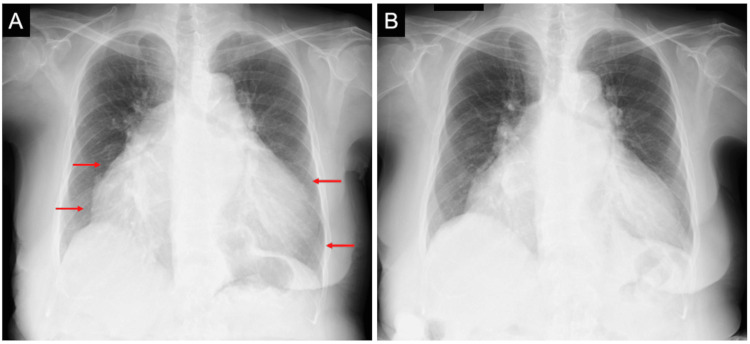
Initial and follow-up chest radiographs. Initial chest radiograph showed a significantly enlarged cardiac silhouette with a cardiothoracic ratio of 85%, mainly depending on right atrial and ventricular dilatations (A, arrows). The follow-up chest radiograph revealed a slight decrease in the cardiothoracic ratio to 80% (B).

A chest radiograph showed a significantly enlarged cardiac silhouette with a cardiothoracic ratio of 85% (Figure 2, Panel A, arrows).

**Figure 3:**
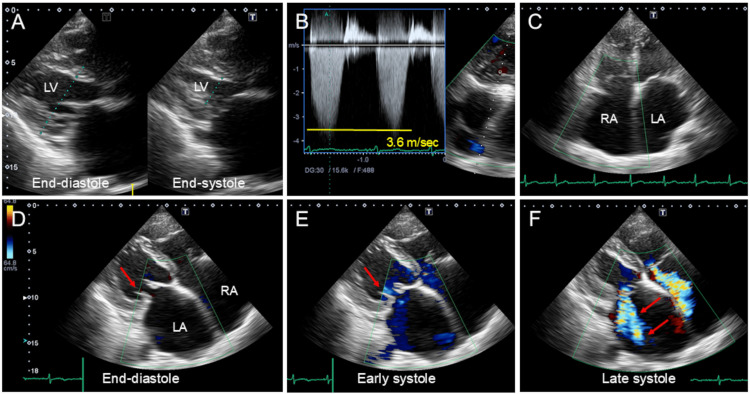
Transthoracic echocardiographic images. A transthoracic echocardiogram showed normal LV systolic function with an ejection fraction of 76% (A). The tricuspid regurgitation pressure gradient was 52 mmHg (B). The LA was markedly enlarged (C). The enlarged LA displaced the posterior annulus onto the crest of the LV free wall. The hamstrung posterior mitral leaflet was tethered (D-E, arrows) and moderate mitral regurgitation with an eccentric jet was seen (F, arrows). Taken together, it was considered to be atrial functional mitral regurgitation related to long-standing persistent AF. The right atrium was also markedly enlarged with moderate tricuspid regurgitation (C-F). LV: left ventricular; LA: left atrial; AF: atrial fibrillation

A transthoracic echocardiogram showed normal left ventricular (LV) systolic function with an ejection fraction of 76% (Figure 3A, Table 2). Doppler echocardiographic measurements disclosed the following values: tricuspid regurgitation pressure gradient of 52 mmHg (Figure 3B), and LV outflow tract velocity time integral of 19.3 cm. The left atrium (LA) was markedly enlarged (Figure 3C), measuring 71 mm in diameter and a volume index of 141 mL/m^2^. The enlarged LA displaced the posterior annulus onto the crest of the LV free wall. The hamstrung posterior mitral leaflet was tethered (Figures 3D-3E, arrows), and moderate mitral regurgitation with an eccentric jet was seen (Figure 3F, arrows) [12]. Taken together, it was considered to be atrial functional mitral regurgitation related to long-standing persistent AF. The right atrium was also markedly enlarged with moderate tricuspid regurgitation (Figures 3C-3F).

**Table 2:** Transthoracic echocardiographic findings.

Variable	Initial presentation	26th week
Left ventricular end-diastolic diameter (mm)	52	50
Left ventricular end-systolic diameter (mm)	29	25
Interventricular septum thickness (mm)	11	11
Left ventricular posterior wall thickness (mm)	11	11
Left ventricular ejection fraction (%)	76	81
Left atrial diameter (mm)	71	71
Left atrial volume index (mL/m^2^)	141	127
Severity of mitral regurgitation	Moderate	Moderate
Severity of tricuspid regurgitation	Moderate	Moderate
Left ventricular outflow tract velocity time integral (cm)	19.3	23.4
Tricuspid regurgitation pressure gradient (mmHg)	52	42

The patient was diagnosed with HFpEF (New York Heart Association (NYHA) functional class III) complicated by impaired ANP secretion and atrial functional regurgitation of the mitral and tricuspid valves due to long-standing persistent AF [13]. She was admitted for the treatment of these conditions.

**Figure 4:**
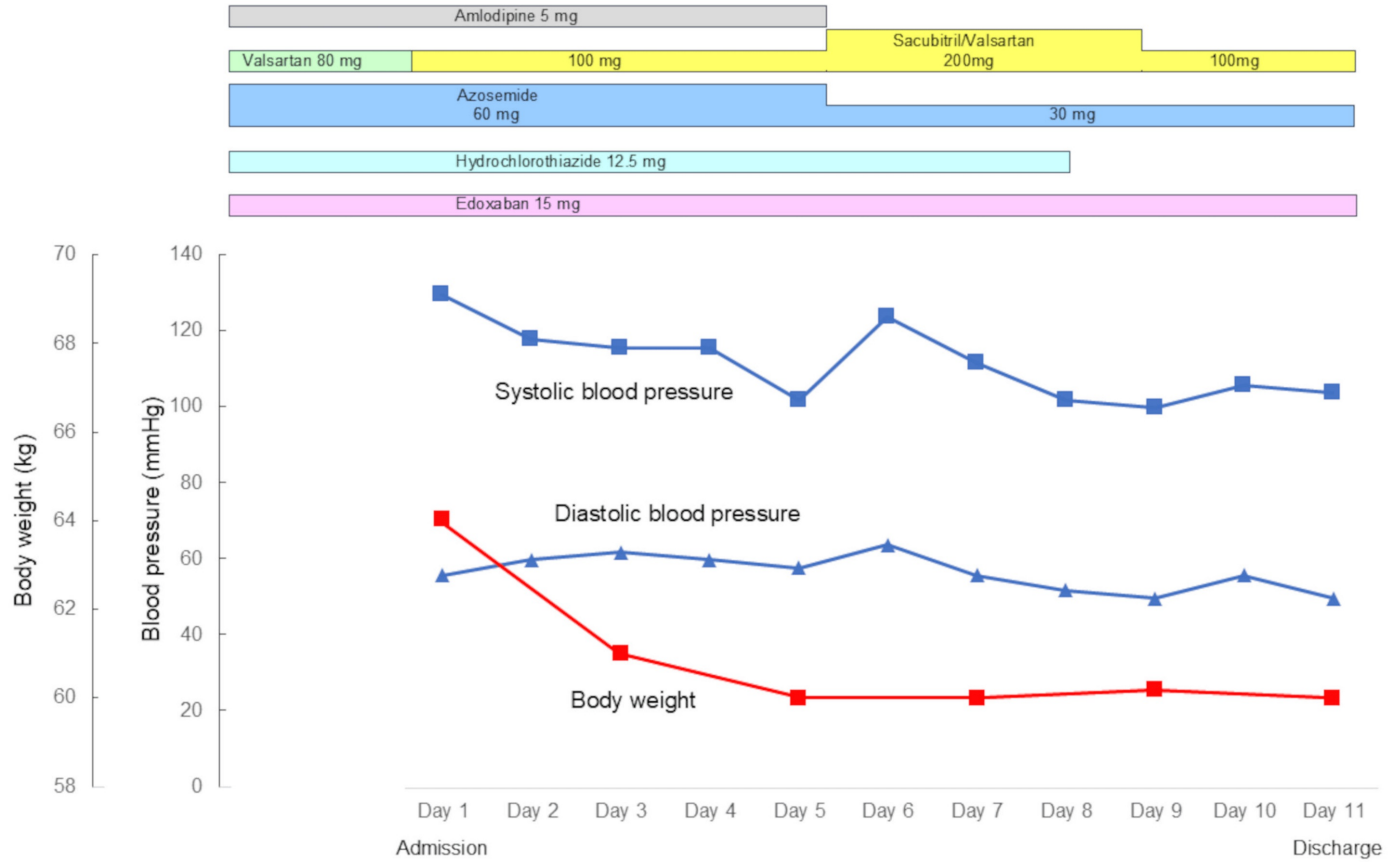
Clinical course during hospitalization. The patient’s weight decreased from 64 kg to 61 kg in the first two days. On hospital day five, her weight further decreased to 60 kg and edema in both lower extremities disappeared. The dose of azosemide was reduced to 30 mg and hydrochlorothiazide was discontinued.

On hospital day one, valsartan (80 mg/day) was replaced with sacubitril/valsartan (100 mg/day) without changing other medications. In other words, sacubitril, a neprilysin inhibitor, was added to existing medications to enhance natriuretic peptide levels. As shown in Figure 4, the patient’s weight decreased from 64 kg to 61 kg in the first two days. On hospital day five, her weight further decreased to 60 kg, and edema in both lower extremities disappeared. The dose of azosemide was reduced to 30 mg and hydrochlorothiazide was discontinued. Cardiac rehabilitation was performed during hospitalization. The patient refused surgical treatment of atrial functional regurgitation of the mitral and tricuspid valves because of concerns about surgical risks at advanced age [14,15]. On hospital day 11, the patient was discharged on sacubitril/valsartan treatment (100 mg/day) after its titration.

**Figure 5:**
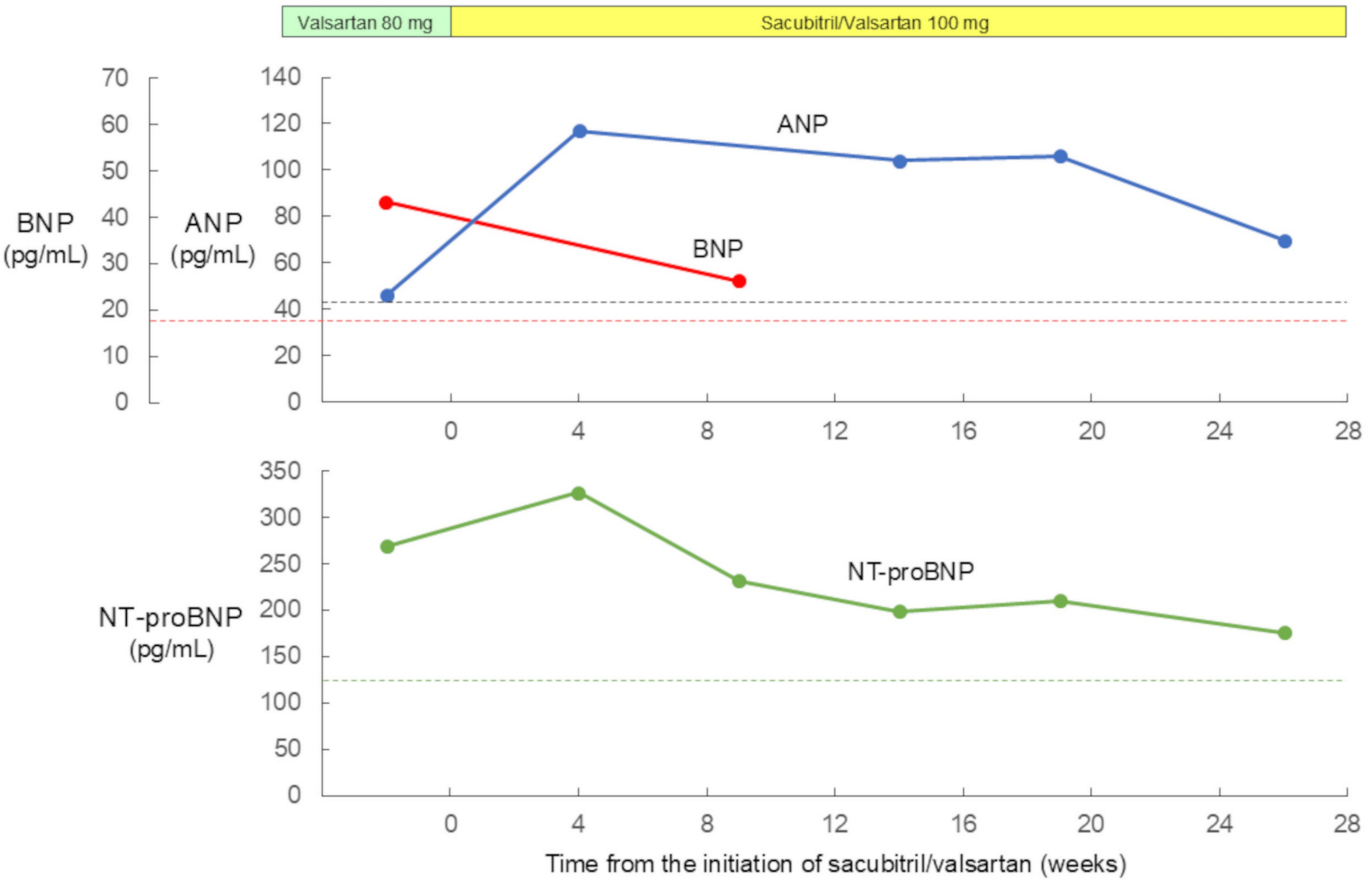
Changes in natriuretic peptide levels during the follow-up period. ANP level significantly increased, more than two-fold, from 46.1 pg/mL to 117 pg/mL in the first four weeks and then remained above 100 pg/mL until the 19th week. It eventually returned to 69.8 pg/mL in the 26th week. NT-proBNP level decreased from 269 pg/mL to 232 pg/mL in the first nine weeks and then remained around 200 pg/mL until the 19th week. It eventually returned to 176 pg/mL in the 26th week. BNP level decreased from 43.3 pg/mL to 26.2 pg/mL in the first nine weeks in parallel with NT-proBNP level. The dotted lines indicate the upper reference limits for natriuretic peptides. ANP: atrial natriuretic peptide; NT-proBNP: N-terminal pro-brain natriuretic peptide; BNP: brain natriuretic peptide

The patient was followed up with medical treatment on an outpatient basis with laboratory tests. Changes in natriuretic peptide levels during the follow-up are shown in Figure 5. ANP level significantly increased, more than two-fold, from 46.1 pg/mL to 117 pg/mL in the first four weeks and then remained above 100 pg/mL until the 19th week. It eventually returned to 69.8 pg/mL in the 26th week. NT-proBNP level decreased from 269 pg/mL to 232 pg/mL in the first nine weeks and then remained around 200 pg/mL until the 19th week. It eventually returned to 176 pg/mL in the 26th week. BNP level decreased from 43.3 pg/mL to 26.2 pg/mL in the first nine weeks in parallel with NT-proBNP level.

Follow-up laboratory tests in the 26th week revealed normalization of hemoglobin, total protein, and albumin levels (Table 1), and a chest radiograph showed a slight decrease in cardiothoracic ratio to 80% (Figure 2, Panel B). A follow-up echocardiogram still showed atrial enlargement with moderate mitral and tricuspid regurgitation. The tricuspid regurgitation pressure gradient was slightly decreased to 42 mmHg (Table 2). The patient remained in stable condition (NYHA functional class II) without recurrence of clinical congestion during the eight-month follow-up period.

## Discussion

In this report, we presented a case of HFpEF complicated by impaired ANP secretion and atrial functional regurgitation of the mitral and tricuspid valves due to long-standing persistent AF. Administration of sacubitril/valsartan augmented endogenous ANP level, which was effective in improving the patient’s symptoms such as dyspnea and edema and reducing NT-proBNP level.

The LA plays an important role in modulating LV filling by acting as a reservoir, passive conduit, and active booster pump during one cardiac cycle [16]. The LA also has an endocrine function, secreting ANP in response to wall stretch and volume overload in the setting of heart failure. These LA functions are impaired in long-standing AF and contribute to the disease progression of heart failure. As for the LA endocrine function, Van Den Berg et al. revealed that the duration of AF was an independent predictor of ANP level, with longer duration predicting lower ANP level [6]. Seino et al. showed that atrial standstill after long-standing persistent AF was characterized by very low or undetectable ANP levels [17].

The atrial fibrillatory amplitude decreases gradually in the natural course of persistent AF [18]. Previous studies have shown that fine AF was associated with longer AF duration, older age, larger LA size, and lower LA appendage flow [19,20]. Atrial functional regurgitation occurs due to atrial dilatation, and in the majority of cases due to long-standing persistent AF. Atrial dilatation leads to the separation of the two leaflets apart forming a coaptation gap in the context of annular dilatation [21]. In the present case, fine AF, enlarged atria, and atrial functional regurgitation of the mitral and tricuspid valves were documented on the initial electrocardiogram and echocardiogram, suggesting degenerative changes of the atria. The fact that the baseline ANP level was not as high as the NT-proBNP level supported this idea and suggested impaired secretion of ANP from the atria.

Previous studies have shown that BNP levels are lower in HFpEF compared with heart failure with reduced ejection fraction, although the precise mechanism remains to be investigated [22]. Matsumoto et al. recently revealed that ANP level was also lower in HFpEF [23]. They demonstrated that HFpEF, presence of AF, and lower baseline ANP level were independent predictors of the greater diuretic effect of exogenous ANP (intravenous carperitide) in patients with heart failure [23]. According to their results, modulation of endogenous ANP levels seems to be a promising treatment, especially in patients with heart failure and impaired ANP secretion. Sacubitril/valsartan can increase endogenous ANP levels through the inhibition of neprilysin [11], as well as intravenous carperitide. In the present case, the patient’s edema was resolved immediately after the administration of sacubitril/valsartan, which allowed for dose reduction of diuretics. Furthermore, follow-up laboratory tests showed normalization of hemoglobin, total protein, and albumin levels, suggesting appropriate volume reduction. Based on serial measurements of natriuretic peptides, it is very likely that these beneficial effects are attributed to the sustained increase in ANP level. The diuretic effect of sacubitril/valsartan may be evident, especially in patients with impaired ANP secretion in response to volume overload. Simultaneous measurement of ANP and NT-proBNP, especially in patients with persistent AF, may help identify such cases efficiently. Further studies in large populations with heart failure are needed to clarify the association between baseline ANP level and the diuretic effect of sacubitril/valsartan. Similar to the time course of NT-proBNP, ANP level eventually declined despite continued sacubitril/valsartan treatment. ANP level is the net result of ANP secretion from the atria and ANP degradation by neprilysin. The final decline in ANP level is explained by appropriate volume reduction, as indicated by NT-proBNP.

Another unique finding was that the ANP level increased in the first nine weeks of sacubitril/valsartan treatment, whereas the BNP level decreased in parallel with the NT-proBNP level. This finding can be explained by the following mechanism. BNP level is the net result of BNP secretion mainly from the ventricles and BNP degradation by neprilysin. Because neprilysin has lower substrate specificity for BNP than ANP, the increase in BNP level after sacubitril/valsartan is much smaller than the increase in ANP [11]. As a result, appropriate volume reduction would have decreased the BNP level, more than offset the impact of sacubitril/valsartan. In the PIONEER-HF trial comparing the in-hospital initiation of sacubitril/valsartan versus enalapril in patients with heart failure with reduced ejection fraction, the use of sacubitril/valsartan reduced NT-proBNP level by 47% and BNP level by 29% from baseline [24]. A recent case report demonstrated that sacubitril/valsartan led to a parallel return to baseline levels in BNP and NT-proBNP in a patient with heart failure with reduced ejection fraction [25]. Our results regarding BNP were supported by these reports. Further studies are needed to determine whether BNP can be used as a biomarker of volume overload in heart failure even in the sacubitril/valsartan era.

## Conclusions

We encountered a case of HFpEF complicated by impaired ANP secretion and atrial functional regurgitation of the mitral and tricuspid valves due to long-standing persistent AF. Administration of sacubitril/valsartan was effective in improving the patient’s symptoms, such as dyspnea and edema, and reducing NT-proBNP level by increasing endogenous ANP level from 46.1 pg/mL to 117 pg/mL in the first four weeks. This case highlights the possibility of impaired ANP secretion in response to volume overload as a predictor of the diuretic effect of sacubitril/valsartan in heart failure. This may lead to individualized treatment for each patient with heart failure based on natriuretic peptide profiles. Further studies in large populations with heart failure are needed to validate the predictive role of impaired ANP secretion.
